# VO_x_/Fe_2_O_3_ Shell–Core Catalysts for the Selective Oxidation of Methanol to Formaldehyde

**DOI:** 10.1007/s11244-017-0873-2

**Published:** 2017-11-03

**Authors:** Pip Hellier, Peter P. Wells, Diego Gianolio, Michael Bowker

**Affiliations:** 1 UK Catalysis Hub, Research Complex at Harwell, Rutherford Appleton Laboratory, Harwell, Oxon OX11 0FA UK; 2 School of Chemistry, Cardiff University, Park Place, Cardiff, CF10 3AT UK; 3 School of Chemistry, University of Southampton, Southampton, SO17 1BJ UK; 4 Diamond Light Source Ltd, Harwell Science and Innovation Campus, Didcot, OX11 0DE UK

## Abstract

Efficient oxidation catalysts are important in many current industrial processes, including the selective oxidation of methanol to formaldehyde. Vanadium-containing catalysts have been shown to be effective selective oxidation catalysts for certain reactions, and research continues to examine their applicability to other reactions of interest. Several VO_x_/Fe_2_O_3_ shell–core catalysts with varying VO_x_ coverage have been produced to investigate the stability of VO_x_ monolayers and their selectivity for methanol oxidation. Catalyst formation proceeds *via* a clear progression of distinct surface species produced during catalyst calcination. At 300 °C the selective VO_x_ overlayer has formed; by 500 °C a sandwich layer of FeVO_4_ arises between the VO_x_ shell and the Fe_2_O_3_ core, inhibiting iron cation participation in the catalysis and enhancing catalyst selectivity. The resulting catalysts, comprising a shell–subshell–core system of VO_x_/FeVO_4_/Fe_2_O_3_, possess good catalytic activity and selectivity to formaldehyde.

**Electronic supplementary material:**

The online version of this article (10.1007/s11244-017-0873-2) contains supplementary material, which is available to authorized users.

## Introduction

The production of formaldehyde (in the form of formalin) is an important industrial process, with millions of tonnes produced globally each year [1]. Formaldehyde has many uses, but is chiefly a precursor to a wide range of higher value compounds, including resins, fertilisers and polymer precursors [1]. It is obtained industrially by the selective oxidation of methanol (via oxidative dehydrogenation, ODH), for which two main catalytic systems exist. One system comprises an oxidic Ag-based catalyst, which, when used at between 500 and 600 °C in a methanol-abundant atmosphere, exhibits 89% yield to formaldehyde [2, 3]. The second system, first reported over 80 years ago by Adkins et al. uses a long-lasting iron molybdate catalyst to generate a formaldehyde yield of 95% under excess oxygen [4]. It operates at lower temperatures in a single step, requiring a temperature no greater than 400 °C to achieve reaction [4]. It is this latter catalyst which is employed in current industrial production; it contains excess MoO_3_ to boost catalyst longevity [5, 6].

Multicomponent oxides, like iron molybdate, are often better suited as industrial catalysts than simpler metal oxides, such as MoO_3_. The presence of a secondary component in an oxidation catalyst can modify surface morphology, aid lattice oxygen movement and boost surface area, all of which benefit catalytic properties [6]. The catalyst lifetime under operating conditions can also be increased. Understanding the nature of the selective surface from analysis of such multicomponent oxides, however, is not tri vial. Through the use of a shell–core catalyst model, elucidation of the surface behaviour is greatly facilitated. Since in a properly formed shell–core catalyst the selective component is confined to the surface layers, techniques which are inherently not surface sensitive may become so; i.e. by confirming that the V exists only on the surface in a shell–core catalyst of VO_x_/Fe_2_O_3_ as employed here (where VO_x_ denotes the surface vanadium oxide-like species), we can be sure that all V spectroscopic signals arise from the surface layers. This is of particular benefit for X-ray Absorption Spectroscopy (XAS), a technique which permits the determination of oxidation states and local structure: since the selective component is at the surface only, XAS analysis greatly assists in understanding surface speciation. Shell–core formation affords a secondary benefit, namely that the resulting catalysts can possess greater surface areas if the core is a high surface area material. The surface areas of relevant selective metal oxides (e.g. MoO_3_ or V_2_O_5_, 5 and 8.6 m^2^ g^−1^ respectively [7, 8]) are typically small, which contributes to their poor catalytic activities when used as unsupported catalysts; by enhancing the surface area of the selective component, catalytic activity can be improved without impairing selectivity. In addition to direct effects on catalytic properties, greater surface areas benefit spectroscopic measurement of the surface. For higher surface areas, there is more surface material present for the same notional monolayer (ML) coverage: consequently, signal-to-noise is improved.

Vanadium oxide catalysts have been widely researched as ODH catalysts for a range of substrates, which includes methanol and smaller alkanes, such as ethane and propane; other vanadium-based oxidation catalysts are well known, such as vanadium phosphate for butane oxidation to maleic anhydride [9–14]. They represent suitable candidates for initial exploration of novel shell–core catalysts, since if shell–core catalysts of VO_x_ can be suitably formed, they can be applied to many different processes. Vanadia itself is selective for methanol oxidation to formaldehyde [15]; however, similarly to MoO_3_ it is poorly active, converting little methanol during reaction. Incorporation into a shell–core catalyst can be expected to improve its catalytic properties considerably. Our initial studies have focussed on methanol oxidation in order to gauge the catalytic properties with respect to formaldehyde production of VO_x_ in shell–core catalysts and demonstrate clear segregation of the catalyst into VO_x_ shell and Fe_2_O_3_ core components.

This shell–core approach has previously been studied in MoO_3_/Fe_2_O_3_ catalysts for methanol oxidation, where it was seen that high selectivity to formaldehyde can be maintained at high methanol conversions [16–20]. The validity of the shell–core model when applied to molybdena-based catalysts was clearly established, in addition to its applicability to the methanol oxidation reaction [18–21]. Mo-based catalysts were investigated since current industrial catalysts for methanol oxidation include iron molybdate, but also because of the high formaldehyde selectivity (though poor activity) of MoO_3_. Using the behaviour of MoO_x_/Fe_2_O_3_ as a guide, we investigated the applicability of the shell–core motif to other metal oxide systems. The surface behaviour and speciation during formation of VO_x_/Fe_2_O_3_ catalysts will be examined, alongside their catalytic efficacies when utilised as methanol oxidation catalysts. The surfaces and structural changes occurring on VO_x_/Fe_2_O_3_ catalysts in relation to calcination temperature and ML coverage will be probed by XAS and complementary characterisation techniques, including Raman, X-ray diffraction (XRD), X-ray photoelectron spectroscopy (XPS) and temperature programmed desorption (TPD).

## Results and Discussion

The VO_x_/Fe_2_O_3_ samples investigated here were prepared by incipient wetness impregnation by adding the vanadate precursor, NH_4_VO_3_, to a sample of α-Fe_2_O_3_, haematite; thereafter, drying and calcination afforded the intended VO_x_/Fe_2_O_3_ catalysts. Ex situ powder XRD measurements show that for calcined VO_x_/Fe_2_O_3_ samples, α-Fe_2_O_3_ is the major phase: the diffraction pattern exhibits α-Fe_2_O_3_ peaks with no FeVO_4_ (Figure S1) [22]. Some other phases of unknown speciation can be seen at low ML coverages, and additional phases are visible for 12 ML VO_x_/Fe_2_O_3_ corresponding to FeVO_4_, albeit of weaker intensity than the haematite peaks. XPS analysis indicates that V remains at the surface; the binding energies of the Fe 2p_3/2_ and V 2p_3/2_ peaks were 710.9 and 517.9 eV respectively, indicating that each element was in its highest oxidation state, Fe(III) and V(V) (Figures S2, S3) [23].

**Fig. 1:**
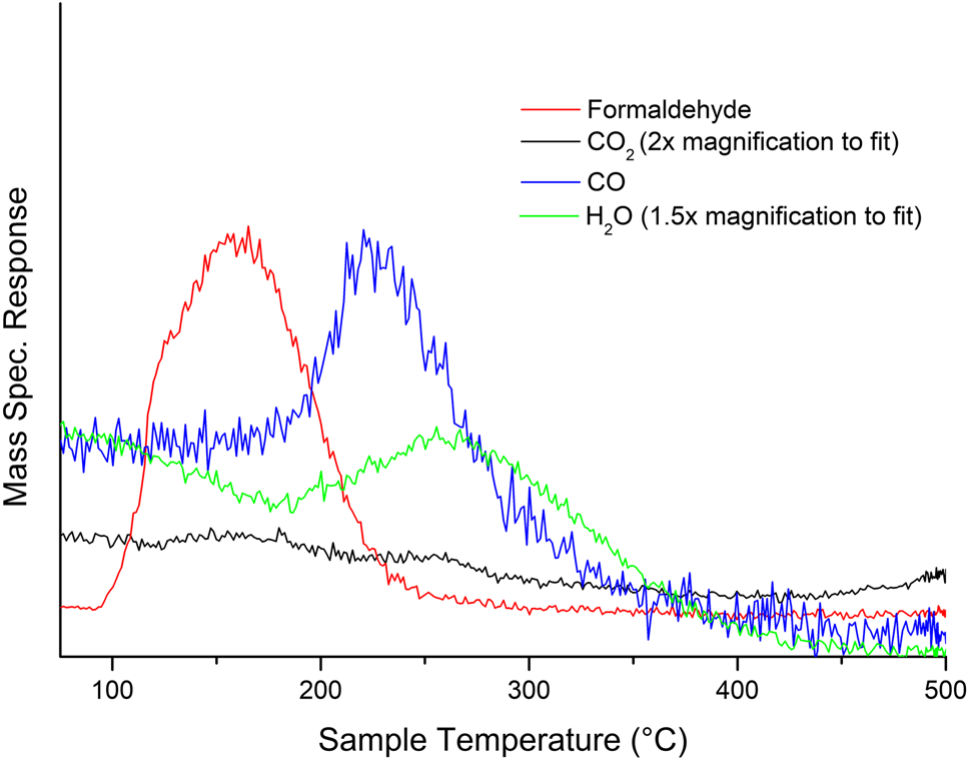
TPD data for 3 ML VO_x_/Fe_2_O_3_ under a helium atmosphere

Temperature programmed desorption (TPD) measurements provide reliable indications of the shell surface integrity for these catalysts. Haematite is a methanol combustor; consequently any exposure of multiple neighbouring core sites (i.e. symptomatic of an incomplete shell) causes combustion. Hence, should formaldehyde be generated in observable quantities, it can be assumed that the iron oxide surface has been modified with V. When analysed in concert with XRD and XPS, TPD measurement of 3 ML VO_x_/Fe_2_O_3_ suggests that segregation is indeed achieved in the catalyst: formaldehyde is observed without concomitant CO_2_ production (Fig. 1).

**Fig. 2:**
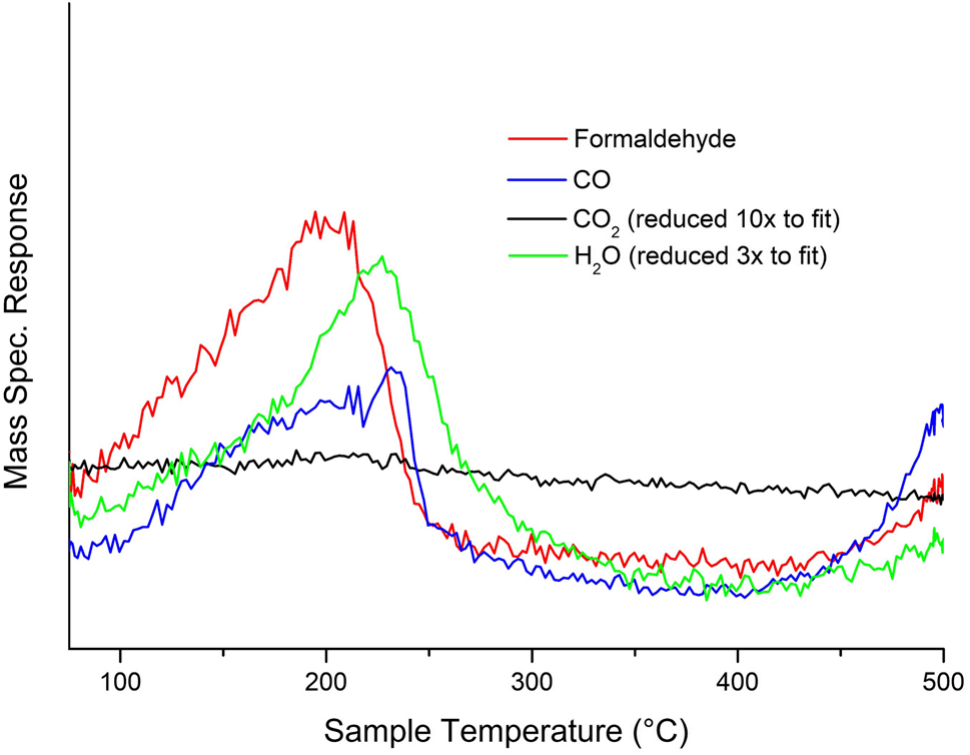
TPD data for V_2_O_5_ after methanol dosing, exhibiting high formaldehyde production (peak at 200 °C), but with some evolution of CO (peak at 230 °C)

For 3 ML VO_x_/Fe_2_O_3_, formaldehyde is produced between 100 and 245 °C, peaking at ∼ 160 °C. This confirms that VO_x_ is present in the surface layers. CO production is observed at higher temperatures, reaching its peak at approximately 230 °C; similar behaviour was observed for previous Mo-based systems, though formaldehyde production peaks at higher temperatures for MoO_x_/Fe_2_O_3_ [18, 19]. It is suggested that isolated cation sites are responsible for CO generation [18, 20, 24, 25]; surface VO_x_ is present in sufficient quantity to preclude multiple neighbouring Fe sites (and the resultant combustion), but isolated Fe sites are still present despite the VO_x_ surface dominance. With a 3 ML VO_x_/Fe_2_O_3_ catalyst formaldehyde production peaks at 160 °C, compared to 205 °C for a bulk V_2_O_5_ catalyst (Fig. 2): the catalytic activity of V_2_O_5_ is demonstrably enhanced through its incorporation into a shell–core structure. For 3 ML VO_x_/Fe_2_O_3_, BET measurements reveal a post-calcination surface area of approximately 18 m^2^ g^−1^, higher than V_2_O_5_ alone (8 m^2^ g^−1^): this can be ascribed to the shell–core process, which is known to enhance surface area [18, 19].

**Fig. 3:**
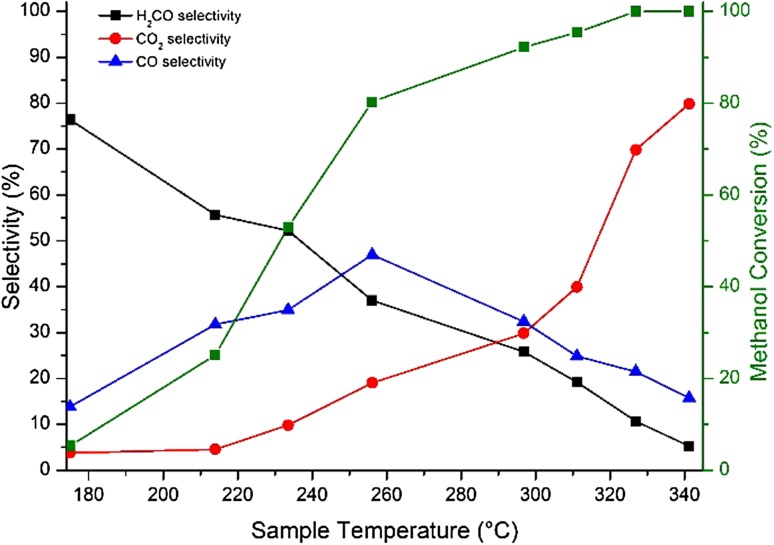
Selectivity/conversion data for 3 ML VO_x_/Fe_2_O_3_; 50% conversion occurs at 230 °C

Selectivity/conversion data from pulsed flow reactions of methanol in a He/10% O_2_ flow afford further understanding of catalytic behaviour (Fig. 3). The initial behaviour is comparable to that seen in TPD, viz. high selectivity to formaldehyde temperatures below 200 °C. However, the selectivity declines with increasing temperature whereas CO shows a peak in selectivity at 250 °C. CO_2_ is produced at higher temperatures and is dominant above 300 °C. This behaviour has similarities to that seen for Mo-based shell–core catalysts reported previously. However, 3 ML VO_x_/Fe_2_O_3_ is a poorer catalyst than 3 ML MoO_x_/Fe_2_O_3_, achieving only 55% selectivity to formaldehyde at 50% conversion compared to 89% selectivity at 50% conversion for MoO_x_/Fe_2_O_3_ [18]. Similar catalytic behaviour is observed upon repeated usage, although studies of prolonged usage and catalyst longevity have yet to be performed. This is unsurprising, since Mo-based systems are preferred for formaldehyde production, but the broadly similar behaviour of VO_x_ in TPD/reaction to that of MoO_x_ further corroborates successful shell–core segregation for VO_x_/Fe_2_O_3_.

**Fig. 4:**
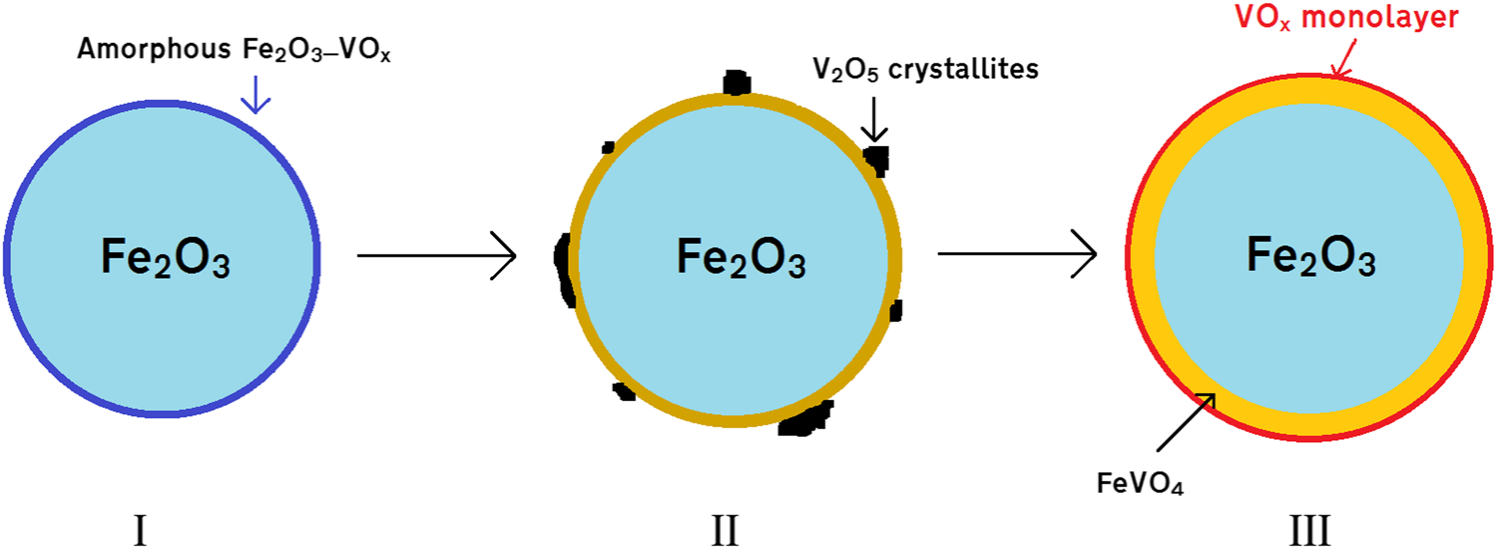
A suggested schematic model of the shell–core formation process as a function of increasing calcination temperature for VO_x_/Fe_2_O_3_ catalysts with > 1 ML coverage: *I* at low temperatures, the surface comprises amorphous VO_x_ units which sit atop a Fe_2_O_3_ core;* II* by 400 °C the surface layer comprises an outer VO_x_ layer, albeit alongside many isolated V_2_O_5_ aggregates;* III* by 500 °C all excess V_2_O_5_ has been converted into the FeVO_4_ sandwich layer, separating the surface VO_x_ layer from the Fe_2_O_3_ core

With TPD and reactivity data confirming that our intended catalytic behaviour is achieved, and XRD indicating that haematite remains the major phase, we can strongly infer that shell segregation has been achieved. It is equally important to understand how segregation occurs in the catalyst and how differences during catalyst synthesis affect the catalytic properties. During the formation of VO_x_/Fe_2_O_3_ shell–core catalysts, we suggest that sufficient thermal energy must be supplied to the surface material to spread it fully across the haematite core to form the shell; we suggest this proceeds via a stepwise mechanism (as described in the text below and in Fig. 4). Similar mechanisms have been proposed for different catalyst systems, corroborating our suggested VO_x_ spreading mechanism [18–20].

**Fig. 5:**
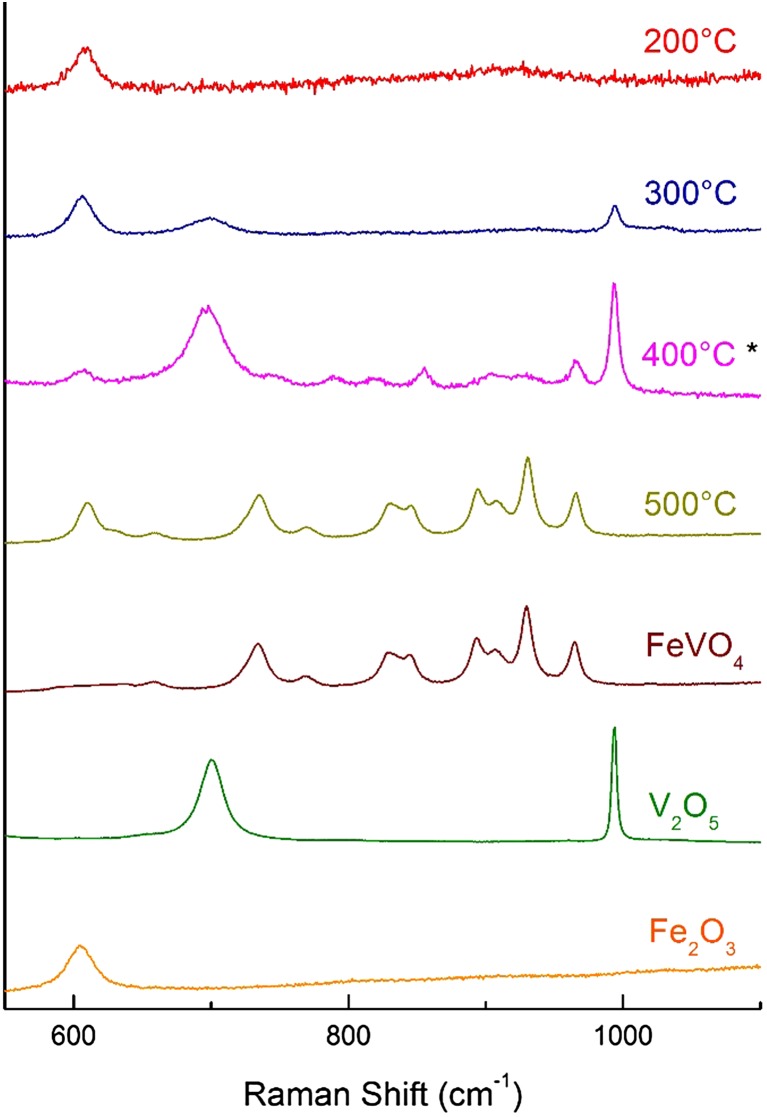
Raman spectra of 3 ML VO_x_/Fe_2_O_3_ calcined at 200, 300, 400 and 500 °C with FeVO_4,_ V_2_O_5_ and Fe_2_O_3_ references (asterisk represents spectrum intensity divided by two to fully include prominent V_2_O_5_ peak at 994 cm^−1^ in spectrum)

Should the temperature be insufficient to achieve this, full shell–core segregation cannot be properly achieved. Incomplete shell species (e.g. V_2_O_5_ aggregates, amorphous VO_x_ or mixtures thereof) are formed in such cases; the temperature attained during calcination affects the product distribution. When analysed by TPD, these “incomplete” catalyst species possess poor selectivity to formaldehyde, producing increased levels of CO and CO_2_. Raman spectroscopy (here under ambient conditions) permits the observation and identification of such species (Fig. 5). Fe_2_O_3_ is observed at 280 and 390 cm^−1^, and V_2_O_5_ at approximately 280 and 490 cm^−1^ (a wider spectrum can be found in the supporting information, Figure S4). V_2_O_5_ also presents two peaks at 701 and 994 cm^−1^, which arise from V–O–V deformation and V=O stretching respectively; it should be noted that these bands are from the crystalline V_2_O_5_ present, and not from the surface VO_x_ species [26]. The latter of these two peaks is pronounced; should V_2_O_5_ be present in other samples, it can be easily detected by this distinct peak.

The presence of FeVO_4_ is evidenced by peaks between 650 and 970 cm^−1^. The sharp peaks at 934 and 969 cm^−1^ emanate from the terminal V–O unit, and the low intensity, broad peaks at 634 and 663 cm^−1^ originate from V–O–Fe bridging modes; V–O–V deformations are not displayed, but occur below 550 cm^−1^ [26]. The peaks situated in between at 738, 773, 845, 860, 895 and 905 cm^−1^ arise from asymmetric stretching modes of VO_4_ [27]. Spectra of 3 ML VO_x_/Fe_2_O_3_ samples calcined at different temperatures demonstrate clear changes in speciation with differences in calcination temperature. At low temperature, calcination is insufficient to form the VO_x_ overlayer or any precursors; a small, broad signal can be seen in the region 800–950 cm^−1^, likely due to amorphous vanadia units deposited from the NH_4_VO_3_ precursor during pre-calcination drying of the catalyst. A summary of the Raman assignments is given below (Table 1).

**Table 1:** Raman assignments for Fig. 1 [26, 27]

Wavenumber (cm^−1^)	Raman assignment (species identity)
610	E_g_ Fe–O stretching (Fe_2_O_3_)
634, 663	V–O–Fe bridging (FeVO_4_)
730–910	Asymmetric VO4 unit stretching (FeVO_4_)
934, 969	Terminal V–O bond stretching (FeVO_4_)
701	V–O–V deformation (V_2_O_5_)
994	V–O stretching (V_2_O_5_)

At 300 °C, sufficient thermal energy is supplied to form small quantities of ordered V_2_O_5_; by 400 °C this process has increased, with significant V_2_O_5_ observed together with the appearance of lesser amounts of FeVO_4_ (as seen from the broad, small peaks between 800 and 900 cm^−1^). After calcination at 500 °C, however, complete conversion to FeVO_4_ is seen, demonstrating that the sandwich vanadate layer of the catalyst has been formed. The apparent lack of V_2_O_5_ at this stage suggests that vanadia surface spreading is fully accomplished by 500 °C, and that no isolated aggregates of V_2_O_5_ remain on the surface. We do not expect the surface VO_x_ overlayer to be visible in Raman here due to limited dimensionality. This behaviour is similar to that exhibited by shell–core catalysts of MoO_x_/Fe_2_O_3_, in which insufficient calcination temperature yields isolated MoO_3_ aggregates at the surface and sufficient heating forms an iron molybdate sandwich layer, akin to the iron vanadate layer [18]. This is highly encouraging, since a fully formed VO_x_ shell atop the core haematite is necessary to prevent direct core haematite participation in the catalysis; haematite has been shown to be a complete combustor of methanol under the conditions used for our experiments [27, 28].

**Fig. 6:**
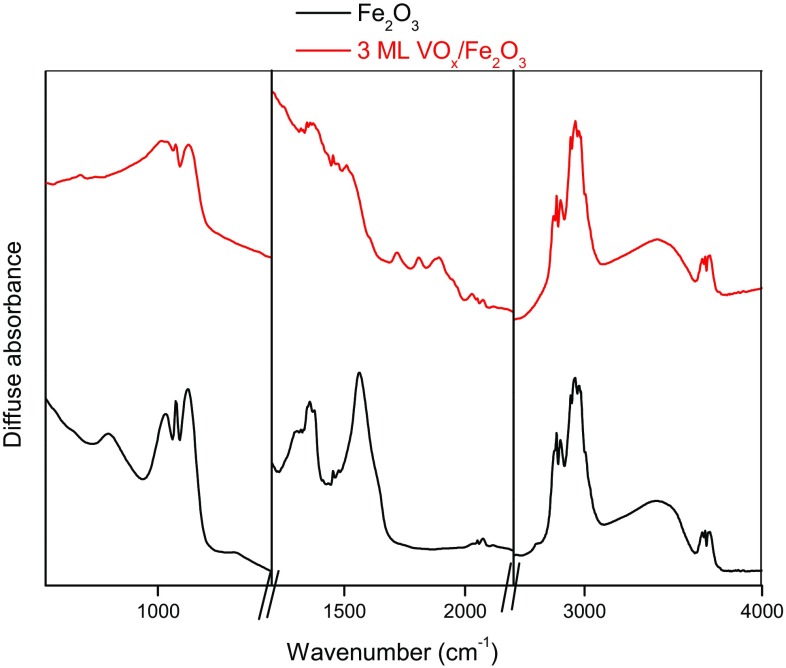
A comparison of key regions in the DRIFTS spectra of Fe_2_O_3_ and 3 ML VO_x_/Fe_2_O_3_ after addition of methanol and temperature ramp to 150 °C. Note: magnification differs between panels to aid clarity

The nature of the surface can be further clarified by Diffuse Reflectance Infrared Fourier Transform Spectroscopy (DRIFTS), which, through the use of a probe molecule, permits surface sensitive measurements to be made. Samples of 3 ML VO_x_/Fe_2_O_3_ and Fe_2_O_3_ were dosed with methanol, heated and examined by DRIFTS (Fig. 6; see Figure S5 for full spectra). Clear differences between the spectra are observed, demonstrating that the surface environment for 3 ML VO_x_/FeO_3_ does not consist of Fe_2_O_3_. For both spectra, adsorbed methoxy species are visible at 2800–3050 cm^−1^ as expected. For 3 ML VO_x_/Fe_2_O_3_, peaks are observed around 1650–2000 cm^−1^; it is suggested in the literature that the peak at approx. 2050 cm^−1^ arises from a V–O overtone corresponding to V^4+^–O. This indicates that V has been reduced to V(IV) from V(V), a process known to occur during oxidation of the methoxy species formed initially on the VO_x_ catalyst [29, 30]. For Fe_2_O_3_, peaks are visible between 1360 and 1560 cm^−1^, corresponding to a formate intermediate present during methanol combustion on Fe_2_O_3_ [31]. Since these are not observed for 3 ML VO_x_/Fe_2_O_3_, it can be assumed that the surface has been successfully modified with VO_x_ to preclude the multiple neighbouring Fe sites necessary to produce formate.

**Fig. 7:**
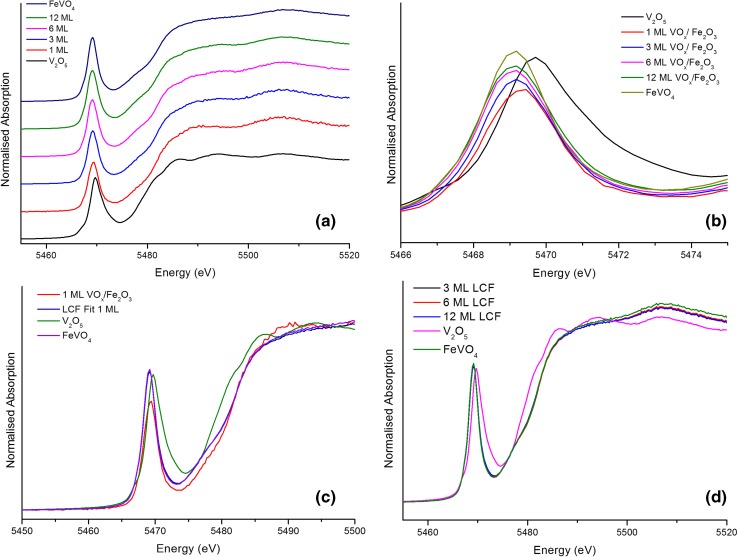
**a** Normalised XANES spectra of calcined 1, 3, 6, 12 ML VO_x_/Fe_2_O_3_, FeVO_4_ and V_2_O_5_; **b** V pre-edge displaying increased peak intensity with increase in VO_x_ ML coverage, alongside the shift in the 1s → 3d peak in the pre-edge region; **c** linear combination fit (LCF) of 1 ML VO_x_/Fe_2_O_3_ using FeVO_4_ and V_2_O_5_ as standards with normalised 1 ML VO_x_/Fe_2_O_3_ for comparison; **d** LCFs of 3, 6 and 12 ML VO_x_/Fe_2_O_3_ displaying similarity to FeVO_4_

XAS measurements have been undertaken on catalysts containing different monolayer VO_x_ coverages on Fe_2_O_3_ (calcined at 500 °C), with FeVO_4_ and V_2_O_5_ used as reference materials. An overview of normalised X-ray Absorption Near Edge Structure (XANES) spectra of the VO_x_/Fe_2_O_3_ catalysts compared to the references is shown below (Fig. 7a). The main XANES transition at the V K edge is a dipole-permitted 1s → 4p transition, but also visible are distinct pre-edge features, arising from dipole-forbidden 1s → 3d transitions (Fig. 7b): these pre-edge transitions feature regularly in vanadium XANES [32]. It is apparent that 3, 6 and 12 ML VO_x_/Fe_2_O_3_ resemble FeVO_4_, which corroborates the model in which further addition of VO_x_ increases the relative abundance of FeVO_4_. The 1 ML VO_x_ pre-edge peak does not relate well to either reference material, occurring at an energy between those of FeVO_4_ and V_2_O_5_. There are noticeable differences in pre-edge peak intensity between VO_x_ ML coverages: we suggest this is likely to be due to the adoption of non-ideal geometries within catalyst shells. It has been reported that the intensity of vanadium pre-edge transitions varies depending on the proximity of coordinating nearest-neighbour ligands to the vanadium centre; the more closely packed the local environment, the greater the intensity of the pre-edge peak [32]. At 1 ML thickness, we suggest that the surface layer structure adopts a somewhat distorted tetrahedral structure; for higher ML coverages, we suggest the surface consists of a V-terminated FeVO_4_ layer, in which V–O bonds are elongated. This affects the local environment around the V, reducing local packing and thus the intensity of the peak. For higher ML coverages, in which a greater proportion of the shell is FeVO_4_, the effect on peak intensity is lessened. Since XAS is an averaging technique, the peak resembles FeVO_4_ more with more FeVO_4_ present in the shell: the effect on peak position/intensity caused by the distorted surface layer is weakened. While this explains the observed behaviour well, it is not possible to dismiss the possibility that the surface comprises a multitude of species: the paucity of shell species in comparison to the rest of the catalyst hinders attempts to characterise them in detail.

Linear combination fitting (LCF) was performed on the XANES spectra using FeVO_4_ and V_2_O_5_ as standards. According to the shell–core model, 1 ML VO_x_/Fe_2_O_3_ should differ from greater ML coverage catalysts due to the absence of the FeVO_4_ sandwich layer. Nonetheless, given that we believe the 1 ML VO_x_/Fe_2_O_3_ structure to be distorted tetrahedral, LCF fitting using tetrahedral FeVO_4_ and square pyramidal V_2_O_5_ standards for comparison will suggest greater similarity to FeVO_4_. This is indeed the case, although the distortion of the structure is sufficient to prompt an unreliable fit (Fig. 7c). Catalysts of greater than 1 ML VO_x_, however, resemble FeVO_4_ (Fig. 7d), corroborating our shell–core model. Given the LCF analysis for 1 ML VO_x_/Fe_2_O_3,_ where its differences from FeVO_4_ can be seen, we suggest that its structure is nominally tetrahedral, albeit somewhat distorted. Similar behaviour, in which V pre-edge intensity decreases with increasing distortion from tetrahedral geometry was observed by Wong et al. [32] which lends support for our suggested geometry for 1 ML VO_x_/Fe_2_O_3_.

## Conclusions

We have probed the applicability of the shell–core model to vanadium-based systems and discovered that:

(i) The VO_x_ shell is sufficiently formed to prevent multiple neighbouring Fe sites participating in the catalysis. TPD data display good selectivity to formaldehyde and minimal CO_2_ production; this suggests minimal exposure of multiple Fe sites at the surface, thereby inhibiting methanol combustion.
(ii) The formation of a complete VO_x_ shell proceeds via observable intermediate phases (viz. amorphous VO_x_, V_2_O_5_ and finally FeVO_4_); this behaviour is very similar to that observed during formation of molybdenum oxide layers on Fe_2_O_3_.
(iii) The resulting VO_x_/Fe_2_O_3_ catalyst is a capable producer of formaldehyde, affording good selectivity and activity. With these results, we are now confident that novel shell–core catalysts on haematite including alternative metal oxides as shell components can be fashioned in a similar manner to those based on V and Mo. Preliminary investigations into other reactions, particularly those for which vanadia-based catalysts are known to be effective (e.g. propane ODH), have been undertaken: in the near future focus will shift to using novel VO_x_/Fe_2_O_3_ catalysts in such reactions.

## Experimental

### Synthesis

3 ML VO_x_/Fe_2_O_3_ catalysts were prepared by incipient wetness impregnation, in which the relevant amount of ammonium metavanadate NH_4_VO_3_ to achieve 3 ML coverage was dissolved in ethanolamine and added dropwise to Fe_2_O_3_ (Sigma-Aldrich, nanopowder, 99%) was obtained as a reference material, while FeVO_4_ was produced by coprecipitation using the relevant amount of iron nitrate nonahydrate [Fe(NO_3_)_3_·9H_2_O] dissolved in water to which ammonium metavanadate in ethanolamine was added dropwise. The mixture was then acidified to pH 2 and the solution boiled to remove liquid. The resultant sludge was removed and dried overnight at room temperature, before being dried further at 120 °C for 3 h. Both V_2_O_5_ and FeVO_4_ were calcined at 500 °C for 24 h before being used as references.

### Catalytic Testing

TPD and pulsed flow reaction data were obtained from a Hiden CATLAB microreactor, comprising a furnace around the sample through which gas passes. For TPD, methanol was injected in microlitre quantities at room temperature, followed by heating to 500 °C under a He flow; for pulsed flow reactions, microlitre aliquots of methanol were injected every 2 min into a 30 mL min^−1^ flow of 10% O_2_/He during a 10 °C min^−1^ ramp to 500 °C. Products of these processes were monitored by the online Hiden QGA quadrupole mass spectrometer during the temperature ramps. Figures using mass spectra data display processed data, i.e. post removal of spectral overlaps.

### Characterisation

Vibrational spectroscopy was primarily used to identify component speciation at different calcination stages and after full calcination at 500 °C. Raman measurements were undertaken using a Renishaw Raman microscope with an 830 nm laser under ambient conditions, with typical measurements ranging between 300 and 1200 cm^−1^ with 1% laser power and 5 accumulations of 20 s each. BET surface area measurements were performed using a Quantachrome Quadrasorb Evo analyser. DRIFTS measurements were performed using an Agilent Technologies Cary 600 FTIR spectrometer with DRIFTS modifications containing the sample holder and the focussing mirrors. XRD was measured on a fifth generation Rigaku MiniFlex benchtop diffractometer with a Cu Kα X-ray source under ambient conditions. V K-edge XAS measurements were performed at B18 at the UK synchrotron Diamond Light Source, Harwell Science & Innovation Campus, Didcot [33]; these used a quick EXAFS (QEXAFS) setup with fast-scanning Si(111) dual crystal monochromator. Each VO_x_/Fe_2_O_3_ sample, V_2_O_5_ and FeVO_4_ were pelletised with some cellulose to aid binding and measured in transmission mode in air using ion chamber detectors with a V foil as reference [33]. Scan duration was typically 3 min per measurement, with three measurements made per sample: these were then merged for analysis. XAS analysis was undertaken with IFEFFIT using the Demeter package (including Athena and Artemis) [34, 35].

## Electronic supplementary material

Below is the link to the electronic supplementary material.

Supplementary material 1 (DOCX 388 KB)
