# Supplementary material for: VOx/Fe2O3 Shell–Core Catalysts for the Selective Oxidation of Methanol to Formaldehyde
Source: Top Catal. 2017 Nov 3;61(5):357–64. doi: 10.1007/s11244-017-0873-2 (PMC6560683; doi:10.1007/s11244-017-0873-2)
Supplement: Supplementary file 1 — Supplementary material 1 (DOCX 388 KB) [file 11244_2017_873_MOESM1_ESM.docx]

Supplementary Information

VO_x_/Fe_2_O_3_ shell-core catalysts for the selective oxidation of methanol to formaldehyde.

Pip Hellier^1,2^, Peter P. Wells^1,3,4^, Diego Gianolio^4^_,_ and Michael Bowker^1,2^

^1^UK Catalysis Hub, Research Complex at Harwell, Rutherford Appleton Laboratory, Harwell, Oxon OX11 0FA, United Kingdom

^2^School of Chemistry, Cardiff University, Park Place, Cardiff CF10 3AT, United Kingdom

^3^School of Chemistry, University of Southampton, Southampton, SO17 1BJ, United Kingdom

^4^Diamond Light Source Ltd, Harwell Science and Innovation Campus, Didcot OX11 0DE, United Kingdom

Figure S1: XRD pattern of 3ML VO_x_/Fe_2_O_3_.


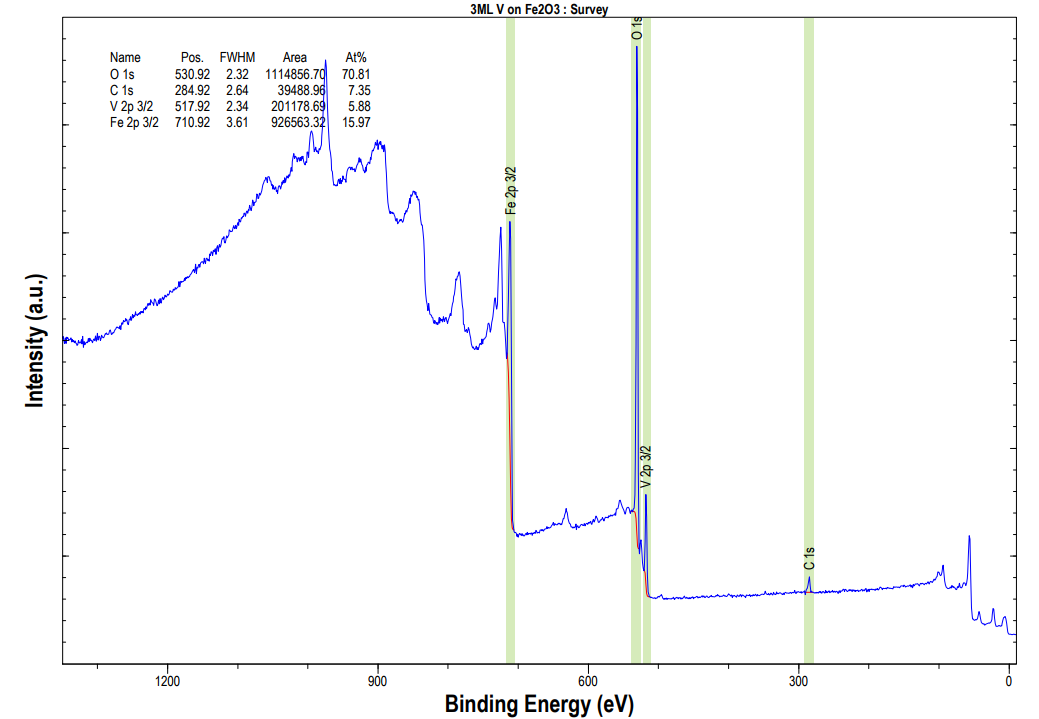


Figure S2: XPS spectrum of 3 ML VO_x_/Fe_2_O_3_.


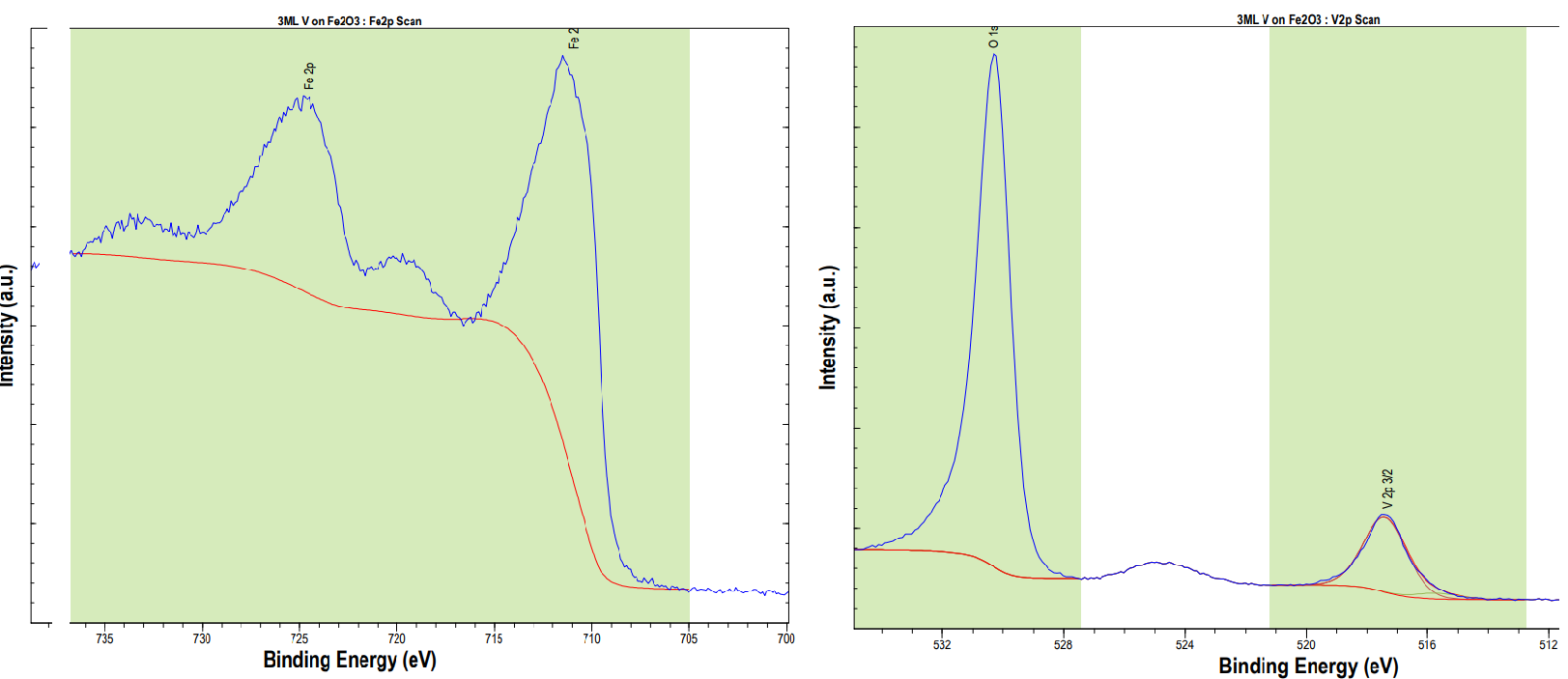
Figure S3: XPS spectrum of 3 ML VO_x_/Fe_2_O_3_ zoomed to display: left) Fe; right) V signals.

Figure S4: Extended Raman spectra of 3 ML VO_x_/Fe_2_O_3_ samples and Fe_2_O_3_.

Figure S5: Full range DRIFTS spectra of 3 ML VO_x_/Fe_2_O_3_ and Fe_2_O_3_.
